# Sexuality and reproduction: contributions of multisituated socioanthropological research with “digital natives”

**DOI:** 10.1590/0102-311XEN229323

**Published:** 2025-05-19

**Authors:** Cristiane da Silva Cabral, Elaine Reis Brandão, Flávia Bulegon Pilecco, Ana Paula dos Reis, José Miguel Nieto Olivar, Daniela Riva Knauth

**Affiliations:** 1 Faculdade de Saúde Pública, Universidade de São Paulo, São Paulo, Brasil.; 2 Instituto de Estudos de Saúde Coletiva, Universidade Federal do Rio de Janeiro, Rio de Janeiro, Brasil.; 3 Universidade Federal de Minas Gerais, Belo Horizonte, Brasil.; 4 Instituto de Saúde Coletiva, Universidade Federal da Bahia, Salvador, Brasil.; 5 Faculdade de Medicina, Universidade Federal do Rio Grande do Sul, Porto Alegre, Brasil.

**Keywords:** Youths, Sexuality, Gender, Reproductive Health, Socialization, Jóvenes, Sexualidad, Género, Salud Reproductiva, Socialización

## Abstract

This study focuses on the processes of socialization for sexuality among youth, addressing dimensions of continuity and change in relation to the Brazilian literature on youth produced during the early 21st century. We interviewed 194 young individuals aged 16 to 24 years, from October 2021 to July 2022, living in different cities and territorial contexts (metropolitan and non-metropolitan areas) in Brazil, composing a socially and culturally diverse set, in terms of ethnicity, social class, gender identity, sexual orientation, with different characteristics and differential access to digital technologies, health care services, and education services. Preliminary results are discussed with first approaches to issues concerning sexuality, sexual initiation in partnership, pregnancy prevention-related difficulties, and reproductive episodes. On the one hand, the results show strong elements of continuity in terms of youth sexual behaviors and moralities in the process of transition to adulthood of young Brazilians today. On the other hand, the empirical research material shows an intense and widespread use of emergency contraception in most researched sites (except Amazonas) and an expressive fluidity of sexual experiences between partners that are not strictly heterosexual - issues to be further analyzed in the next years in order to shed light on the generational peculiarities of this process.

## Abstract (es)

Este artículo se centra en los procesos de socialización para la sexualidad entre los jóvenes y destaca las dimensiones de continuidad y cambio respecto a la literatura brasileña sobre la juventud de principios del siglo XXI. Se entrevistaron a 194 jóvenes de entre 16 y 24 años de edad en el período entre octubre de 2021 y julio de 2022, que viven en diferentes ciudades y contextos territoriales (capital e interior) del Brasil y que componen un conjunto social y culturalmente diverso en cuanto a clase racial, identidad de género, orientación sexual, con diferentes marcas y acceso diferencial a tecnologías digitales, servicios de salud y educación. La discusión de los resultados preliminares incluye los primeros acercamientos al tema de la sexualidad, la iniciación sexual en pareja, las dificultades relacionadas con la prevención del embarazo, así como los episodios reproductivos. Por un lado, los resultados muestran sólidos elementos de continuidad en términos de moralidad y comportamiento sexual de la juventud en el proceso de transición a la edad adulta de los jóvenes brasileños de hoy. Por otro, en el material empírico de la investigación se constata un uso intenso y generalizado de la anticoncepción de emergencia en la mayoría de los lugares investigados (excepto Amazonas) y una fluidez expresiva de las experiencias sexuales entre parejas no estrictamente heterosexuales, temas que se analizarán más a fondo en los próximos años, con el fin de iluminar las peculiaridades generacionales de este proceso.

## Introduction

The process of building the self and transitioning to adulthood is marked by the intensification of socialization for sexuality in adolescence and youth, comprising the learning of gender norms and expectations that inform the behaviors of boys and girls. This is the general assumption that underlies the research conducted between 2021-2022: *Young People in the Digital Age: Sexuality, Reproduction, Social Networks, and Prevention of STIs/HIV/AIDS*. The research included young individuals aged 16 to 24 years, cisgender men and women, but also trans people, from different social backgrounds according to the markers of gender, social class, skin color/ethnicity, reproductive experience, living in different regions of the country. We interviewed young individuals from different cities so as to represent the diversity of youth socialization and sociability processes in metropolitan and non-metropolitan urban contexts. This undertaking aims to produce comparative assessments between studies on youth sexuality and reproduction of the early 2000s and the current historical-political-social context.

We observed a widespread use of the internet and social interaction involving social networks. Undoubtedly, computers and smartphones have provided more than a technological revolution; there is an argument for a “social revolution” through the rise of new relationships between people and the setting ^1^ ^,^ ^2^. “Social networks” and their current forms of connection via the internet emerge as important agents in the current context of youth sociability and socialization, promoting diffusion, reification, contestation, modulation of diverse behaviors, values and practices. Through them, certain events occur and quickly connect people that had been previously separated.

Youth have undergone profound transformations in the world of work, which has become more precarious and informal, permeated by intense state violence (especially police), ethnic, racial and gender discrimination, at a time of rising extremist far-right views in the international and national context, with strong impacts on the public debate on sexuality and intensification of gender inequalities. Youth sexual behaviors are marked by upsurging conservative morals in the national and international socio-political context ^3^, which has promoted a continuous suppression or dismantling of initiatives for education on sexuality, gender and rights and programs for prevention of sexually transmitted infections (STI)/HIV/AIDS and pregnancy in schools ^4^ ^,^ ^5^.

This study aims to understand the sexual and reproductive behaviors of young Brazilians, their contexts and developments. We addressed possible specificities of the current generation, with special interest in the protection practices (or lack thereof) in the face of the “dangers arising from sex” ^6^ ^,^ ^7^. Reflection on how contemporary “risks” associated with sexuality are managed in everyday life ^8^ ^,^ ^9^ is relevant, seeking to understand the logics underlying the adoption or not of a method in a given context, both as to social-cultural aspects and life course. Seemingly, the effects of the conservative moral crusade and the dismantling of various public policies in the area of health and education are already observed in the results obtained. We opted for the use of gender-neutral language in cases of gender-specific words. This seeks to address the political dimension of language, so as to discontinue (or, at least, make explicit) the traditional universalization of subjects by language in the masculine form.

## Methodological aspects

This is a socio-anthropological and multicenter study, with 194 individual interviews using a semi-structured guide, including young individuals aged between 16 and 24 years, adopting the biographical theoretical-methodological approach to analyze contexts, elements and repercussions of certain affective-sexual-reproductive events of those involved ^10^.

The research was conducted in four capitals - Porto Alegre (Rio Grande do Sul State), Rio de Janeiro, Salvador (Bahia State), São Paulo - and in two non-metropolitan cities of the country: Conceição do Mato Dentro, in Minas Gerais State (23,000 inhabitants) and São Gabriel da Cachoeira, in the state of Amazonas (approximately 51,000 inhabitants). With this diversity (quota of 40 interviews per capital and 20 interviews in each non-metropolitan city), we sought elements characterizing values, sociability and experiences according to regional contrasts, addressing possible contrasts between metropolitan and non-metropolitan cities.

We intentionally composed the set of participants according to the criteria of quotas by sex/gender, social class and reproductive experience before the age of 20 years, an age defined by the World Health Organization (WHO) and the Brazilian Ministry of Health to identify pregnancies/parenting experience in adolescence. We sought diversity in terms of sexual orientation and racial identity, with a view to understanding important modulations in sexual and reproductive biographies based on intersectional dimensions such as skin color/ethnicity, social class, sex and gender identity, region, among other social markers of differentiation.

In order to enable a generational approach in the production of empirical data, we selected and trained young researchers, undergraduate or graduate students in Public Health or Social Science programs. An interview is a social relationship, established in the encounter between two intersubjectivities ^11^. There is no control of this interaction, but the necessary elements can be favored for a good quality of this encounter - always unique.

The strategies for disseminating the research and approaching potential participants included the mobilization of the network of personal and professional relationships of the researchers involved, posting of informative texts in WhatsApp groups and other social networks (Facebook and Instagram), electronic form for identifying young individuals interested in giving interviews, in addition to the use of the snowball technique. Fieldwork was performed between October 2021 and July 2022, during the period of mitigated COVID-19 pandemic, with the possibility of returning to in-person activities at the universities involved in the study. The interviews were held mostly in person and in a place chosen by the participant. All interviews have field notes, containing a description of where and how the respondent was approached, the negotiation and conduct of the interview itself, facility, difficulty and general perceptions about the interaction with respondents.

The search for young people willing to talk about their affective-sexual trajectories was difficult in all metropolitan areas, especially among men. The quotas of female respondents were the first to be met, especially those from low-income strata. Conversely, middle-income male rerspondents with some reproductive experience were hard to find. Especially in São Gabriel da Cachoeira, it seems to have been even more difficult to be able to talk about sex: several young persons refused the invitations, others interrupted the interviews, expressing more ostensive discomfort or denial to talk about issues related to sexuality with strangers.

In summary, the set of young persons interviewed (n = 194) comprises cis women (n = 100) and cis men (n = 87), but also trans people (male and female) and non-binary people (n = 7). More than half of them self-declared as black or brown (n = 119); almost a third are LGBTIA+ (n = 58). There is an important presence of young people without religion (n = 89), but also of catholics (n = 25) or evangelicals (n = 30), an interesting aspect for future reflection on the effects of adopted religion on the sexual and reproductive behavior of this generation. There is a large number of young people with secondary education and only five young people with very low education level. Certainly, they exist in large urban and non-metropolitan areas, but they are increasingly fewer as a result of the successive government effort to expand education in general and eliminate illiteracy in the country. This low education profile should also be considered as a reflection of the strategies used to invite research participants (online invitation and snowball technique).

We recorded and transcribed the interviews, which were reviewed by the respondents. We produced analytical summaries and synthetic charts of the biographies as an auxiliary strategy for managing the large volume of empirical material. We use the NVivo software (https://www.qsrinternational.com/nvivo/home) as a support tool for organizing, categorizing and storing the content produced in the six sites. The study was approved by the Brazilian National Research Ethics Committee (CONEP, acronym in Portuguese) and the research ethics committees of the coparticipating institutions (University of São Paulo: CAAE 46977021.3.0000.5421; Federal University of Rio de Janeiro: CAAE 46977021.3.3005.5286; Federal University of Bahia: CAAE 46977021.3.3003.5030; Federal University of Minas Gerais: CAAE 46977021.3.3001.5149; Federal University of Rio Grande do Sul: CAAE 46977021.3.3002.5347). All respondents signed the Assent and Informed Consent Form (parental consent was waived for children aged < 18 years) or the Free and Informed Consent Form (young people aged 18 to 24). Fictitious names are used in this publication.

## Results

The joint analysis of the 194 interviews is a challenge. In this article, we provide an initial overview of some issues to understand certain changes and permanence in the experience of sexuality of youth born and socialized in this period of interactions through social networks and digitalization of daily life ^12^ ^,^ ^13^. There is special interest in the process of socialization for sexuality and preventive behaviors (or lack thereof) in relation to contraception and reproduction. The structuring axes of the results presented are ^14^: agents of socialization for sexuality and the beginning of sexual life in partnership; the permanence of contraceptive difficulties; and reproductive experience: pregnancy and abortion episodes.

### Agents of socialization for sexuality and the beginning of sexual life in partnership

Socialization for sexuality comes not only with the first information about sex, pregnancy and STI prevention methods, but also with conversations about first kisses, dating, erotic experiences with one’s own body and sexual experiences with partners. It is a process that intensifies with perceived bodily changes within the family, school or peer groups ^15^.

In general, young people report the school as the place where they obtained the first information on topics related to sexuality. Criticizing, they pointed out that the contents taught, eminently during elementary school, were centered on biological aspects, reproductive system, and STI/HIV/AIDS prevention methods, an unsatisfactory approach in view of what they wanted to receive. However, the sexual and gender violence issue seems to have entered the school setting because several accounts emphasized the importance of discussions on the identification of cases of sexual abuse and violence. Despite the criticism, educational institutions appear as safe spaces for questioning compulsory heteronormativity.

Other information sources are mentioned as means to approach the sexuality issue: television and films; internet (such as Google) or through influencers on Instagram and TikTok; funk music; erotic magazines; catechism classes or church youth groups, among others. Most young people reported casual access to pornography, in situations such as receiving unsolicited content through social networks; but there is also search for such content on the internet. These reports appeared more frequently in São Paulo, where young people indicated watching pornographic materials individually or collectively.

The moral panic that educational managers and institutions often have about the possible unrestrained access of young people to pornographic content through the internet is not supported in the interviews. On the contrary, the young people speak critically about these contents: some indicated that, after satisfying their curiosity about sex, they stopped accessing porn videos because they did not consider it “healthy”, for lack of interest or for deeming that pornography provides a stereotypical view of sex and bodies. A young man from Salvador (20 years old, middle class) said that “*pornography is a sex different from reality*” and harmful to sexual practice with real partners. This type of position has been found in other contexts ^16^.

Few indicated the family as the source of the first pieces of information about sexuality. The subject is still considered taboo and, when addressed, female figures such as mothers, cousins, sisters are those who speak from the perspective of the risk of pregnancy (for women) or STI (for boys). Silencing seems to involve the fear that dialogue on the subject is a means of encouraging sexual relations, resulting in early reproduction and a deviation in the process of transition to adulthood. This is a characteristic that remains indelible in more than twenty years of research on youth and sexuality, as the result is similar to other studies from the early 2000s ^17^ ^,^ ^18^ ^,^ ^19^ ^,^ ^20^. In Conceição do Mato Dentro, a respondent from an evangelical family reported that, when she was 11 or 12 years old, a teacher gave her a science book in which the topic of sexuality was addressed. On that occasion, her grandmother tore up the book, which made her feel very ashamed. Thus, she concluded that the family was not a good source of information/dialogue. Few respondents reported talking to male figures about the topic. A boy from Salvador indicated that his mother used to give him advice, while his father was “*sexist*”, as he defined it, and encouraged him to have sex early, not worrying about what could happen to his partners: “*he* [the father] *said ‘you really should get laid with these chicks, put a condom on, mess around!’*”.

Sexual initiation with a partner is one of the main events in the process of socialization for sexuality in youth ^21^. An expected event, which is not always planned, the first sexual intercourse occurred between the ages of 14 and 16 years. Girls, mostly, had their “first time” with older or same-aged boys; in the case of boys, it occurred mainly with same-aged girls. In Rio de Janeiro and Salvador, young men also mentioned, with some frequency, sexual initiation with slightly older women with more sexual experience. Overall, they identified their partners as belonging to the same racial group, with few exceptions.

The specificities of sexual initiation of LGBTIA+ people (n = 58) are not covered in this article. However, it should be noted that the first sexual intercourse of these young people includes both opposite-sex and same-sex partners (in this case, 6 women and 9 men). Also absent are the particularities of young virgins (n = 28): they are between 15 and 23 years old, mostly without religion (n = 15), cisgender people (n = 27).

The first sexual intercourse is mostly that which involved penetrative sex, usually described as uncomfortable and bad. A young woman (São Paulo) indicated that the intercourse had not been pleasurable, stating that “*it was as if I were there only to give pleasure and not to receive it*”. Olivia (Conceição do Mato Dentro) describes her sexual initiation as “*traumatizing*”, the result of an impulse. Several young women in Rio de Janeiro reported that they felt pain, were pressured or that their partner was “*sexist*”. A young man (Rio de Janeiro) who had his first intercourse with his girlfriend said that she had a higher expectation than what it actually was: “*she expected wow and it was just sex*”.

There are also reports of sexual initiation as something good and satisfactory. Luana (20 years old, low class, Salvador) enjoyed her first sexual intercourse, despite it not being “*romantic*”. She was 15 and her partner 18 (he is her current partner and father of her daughter). The intercourse occurred at his house when his family was out, the mother was working and the younger sister was at a cousin’s house: “*It was nothing forced... it was out of curiosity and desire*”. Although most of the young people classify the first sexual experience as poor, the following experiences, with partners with whom they have more intimacy, are usually reported as better, as they begin to learn about pleasure and know their body and that of their partner better.

Sexual initiation is mostly described as something that “*happened*”, with characteristics of a certain type of spontaneity, an aspect previously indicated as structuring the initial sexual life of young Brazilians ^21^. Although a minority, there are cases with prior planning, something that was “*prepared piecemeal*” in partnership. It takes place in the household setting, in the home of one of the two people involved. Some young women (Conceição do Mato Dentro and São Paulo) reported that the first time was previously discussed with their partner, in a relationship of “*trust*” and “*affection*”. In Conceição do Mato Dentro, girls who reported they had planned the event had their sexual initiation at a slightly older age than the other girls from the same site, who assessed their sexual initiation more negatively.

The language employed reveals the sexual and gender morality that organizes sexual interactions. Despite the various changes related to the autonomy of bodies and female sexuality, traditional terms still remain to describe the sexual initiation of girls. For example, in Rio de Janeiro, some young women from the low-income strata referred to the first experience as having “*got lost*”: “*As soon as I got lost, I told my mother*”. In São Gabriel da Cachoeira, the term used is “*preserve oneself*” to designate the context in which, after the only sexual intercourse she had, around 14/15 years old in her house with a “*date*”, the respondent said that she had “*preserved herself*” again, to continue her education. In a context, losing one’s virginity expresses a moral connotation that subtracts from the young woman some attribute of value, which she would have lost by “*giving herself to her partner*”. In the other context, preserving oneself seems to refer to a life strategy and attempt to control reproduction in view of lack of preparation or resources to avoid pregnancy or STIs.

### The permanence of contraceptive difficulties

Contraception remains a responsibility of women, even in a context in which the use of external prophylactics was repeatedly mentioned. The young women mainly indicated the internet, with google searches, as a source of information on contraceptive methods. Some girls watched videos on Youtube; others said they followed gynecologists’ profiles on Instagram, following content on contraception. In the case of men, there was a general lack of knowledge about female contraceptives and their mechanisms of action.

External prophylactics and coitus interruptus are used interchangeably with hormonal methods as strategies to prevent pregnancy. Internal prophylactics occasionally appeared in the reports of young people from São Paulo and Rio de Janeiro. However, the condom remains a nuisance artifact for this generation. Avoidance and discomfort appear in the accounts of sexual initiation and in subsequent intercourses. Despite the difficulties, the boys were mostly responsible for obtaining the product, in private drugstores and, to a lesser extent, in basic health care units.

Among hormonal methods, contraceptive pills predominate at some point in the sexual trajectories of young women. The injectable contraceptive, mostly used by girls from lower classes, alternates with other behavioral methods (coitus interruptus; table) or barrier methods. It was difficult to observe any regularity in the use of contraceptive methods; the alternation of partners contributes to this inconstancy, as well as the search to find a satisfactory method. Young people under the age of 18 years reported difficulties in accessing methods other than external prophylactics, situations in which adult supervision was required (improperly) during gynecological care, or even lack of money to access other contraceptive methods in private drugstores.

Forgetfulness in obtaining more contraceptives and lack of knowledge of correct use are involved in some unforeseen reproductive experiences. It is impressive to find, in the current generation, reports commonly obtained decades ago. For example: a young woman from Salvador (20 years old, pregnant at the age of 16 years) decided to start taking the pill, the same one her friend’s mother used. However, the girl misused both the contraceptive and the morning-after pill: “*Every time I had a relation I would take a medicine, and I thought it was ok. Then I started taking the morning-after pill, I didn’t take it every day, but I took it very often*”. Another young woman (São Gabriel da Cachoeira) reported that her mother instructed her to take a “*vaccine*” to prevent pregnancy; however, she sometimes used a condom and sometimes took an oral contraceptive. She was afraid that something would happen to her body and stopped taking the “*medicine*”; she was pregnant at the time of the interview.

The relationship with health care professionals, based on the accounts of the young women, leaves much to be desired from the point of view of the necessary care, dialogue on contraceptive methods, and clarification of doubts. In general, the young women talk to friends about contraception. Schools and health care services appear very little as spaces for debate or as a source of information on the subject.

Most of the young women are not satisfied with the contraceptive methods in use and wish for a method that provides them with greater comfort and fewer side effects. The number of symptoms and side effects caused by hormonal methods is quite significant, significantly impairing the daily lives of the young women. There are several complaints of excessive bleeding, headaches, swelling/edema, weight gain, vascular problems, malaise, indisposition, and decreased libido by those using birth control pills or injectable contraceptives. The alternation between types of methods was quite frequent, with a composition between them throughout the youth trajectories. These attempts to adjust to some protection that is less iatrogenic lead to the possibility of unforeseen pregnancy.

It is worth mentioning the presence of information in São Gabriel da Cachoeira on “*blessings*” and ritual processes associated with menarche and pregnancy prevention in the accounts of young people in this city. In conversation circles held during fieldwork, several young women consistently explained the importance - and effectiveness, when “*well done*” - of these ancestral procedures, which are not only meant to prevent pregnancy, but to take care of women's bodies, lives, knowledge and reproductive possibilities.

### Reproductive experience: pregnancy and abortion episodes

The search for participants with some reproductive event background was intentional. We expected to gather 100 people (half of the empirical universe) but there was much difficulty in identifying young people with this profile. We managed to interview 83 people, with 98 reproductive episodes, the vast majority reaching birth. We recorded only 11 abortions (5 provoked). There are 15 respondents with more than one pregnancy.

In general, the reports indicate pregnancies that occurred unexpectedly. The planning contexts allude to pregnancy as a strategy to leave the parents’ home and/or move away from the family of origin (marked by strong social vulnerability). A 17-year-old women, black, from a low-income class, resident of Porto Alegre, considers that her life is easier now that she is a mother and resides with her husband. Before, she took care of her two younger sisters for her mother to work, and she believes that she already had a motherhood experience during this period: “*mother of my sisters*”. In general, the respondents reported that there was an understanding between the partners about maintaining the pregnancy. Most of the young mothers said they had the support of their partner and family during pregnancies and after childbirth with child care work. Cohabitation after the discovery of pregnancy or childbirth was frequent in almost all cases, although in some cases a subsequent separation occurred.

Many respondents said they had thought about having an abortion. However, factors such as the illegal status of the procedure, lack of support from partner and family, and religious and moral issues seemed to influence the decision to continue the pregnancies to birth. Laíz (Conceição do Mato Dentro), 14 years old at the time, low class, Catholic, brown, reports that she wanted to end the pregnancy as soon as she discovered it, but she heard that she “*would have to admit her mistake*”. A young woman from Rio de Janeiro was forced by her mother to continue the pregnancy. In Rio de Janeiro, only one girl and one boy talked about end of pregnancy. In both cases, the girls’ families knew about the pregnancies and supported the abortion decision; the boy’s family was not aware of the pregnancy. A young woman in São Paulo, pregnant at the age of 18 years, decided to have an abortion with the support of her partner, who bought the drug Cytotec through Facebook. The procedure occurred at his house. In São Gabriel da Cachoeira, one respondent says that, given the suspicion of pregnancy due to a menstrual delay, she ingested large amounts of tea and her “*menstruation went down*”. In her words, “*boldo tea saves a lot of people*”.

## Discussion

Current empirical data and resulting reflections on socialization for sexuality, sexual initiation (heterosexual partnerships), contraceptive practices and reproduction, including abortion, among young people aged 16 to 24 years, have as their main counterpoint the productions of the early 2000s about young Brazilians ^18^. This is an analytical strategy that seeks to reflect on possible changes and permanences/continuities regarding youth sexual and reproductive behaviors and moralities, after two decades of transformations in the sociabilities of youth, permeated by drastic political, economic, and environmental changes, and digitalization of daily life ^12^ ^,^ ^13^ ^,^ ^16^.

This generation of youth, sometimes called “digital natives”, permeated by the consolidation and over-massification of online communications, was also challenged by the occurrence of the COVID-19 pandemic. Research and youth lives were affected by the first major health emergency of the 21st century. Many young people had to deal with the fear of infection, greater/lesser parental control and invented alternatives to recover sociability in the period immediately prior to the fieldwork.

Sexual initiation continues to be marked by a certain spontaneity ^21^, with few cases of planning, especially as to contraceptive methods, at the first sexual intercourse. Sexual morality in terms of “*loss of virginity*” remains present in the current generation. The first sexual intercourse continues to be considered mostly that in which there is penetrative sex ^22^.

Old and new actors inhabit the context of learning sexuality. Family and school remain important agents in the process of socializing contraceptive and preventive practices: mothers are a reference for girls and boys, even if the conversations are not frankly open and have “*be careful*” outlines; fathers remain absent from this support. Schools, for their part, continue to inconsisten with youth expectations. Issues related to sexuality are sidelined in school contents, addressed in the form of individual accountability and risk (sex as a danger) in several schools in the different cities studied.

There are new actors on the setting: the internet is a source of diverse information for young people, including about sexuality. It multiplies the possibilities of sociability and access to the most diverse information and knowledge. As “digital natives”, which certainly does not apply in the same way in all contexts, these young people learn to navigate the world they inhabit, producing paths and knowledge in their processes of socialization for sexuality.

A considerable difference among the youth in the cities studied was the strong refusal to talk about sexuality and reproductive health in São Gabriel da Cachoeira, which led to difficulties in conducting interviews on certain topics and details requested. However, this refusal does not seem to be related only to the context of the interview. Conversations between young people and their mothers usually deal with the context of menarche, “care” to be adopted during the menstrual period. Dialogue on sexual desires and practices is possible among peers, in intimate, protected and everyday contexts, particularly among young women ^23^. Ethnographic observations suggest that within the local dynamics, talking about sexuality in cross-gender relationships and, especially, with age difference, is an act that can be directly connected with the dynamics of sexuality and conjugality: the scene of a man “*talking*” with the girl or her father can imply openness to sex and marriage ^23^ ^,^ ^24^ ^,^ ^25^.

It is not part of the scope of this article to analyze the processes that have led to decreased adolescent fertility rate in recent years ^26^ ^,^ ^27^. However, it was felt very strongly in our research because, as pointed out, it was difficult to find young people who had bore children before the age of 20 years, especially middle-class young men. We believe that the widespread use of emergency contraception among respondents is contributing to a lower occurrence of unexpected pregnancies. This view is reinforced by data from the last *Brazilian National Survey of School Health* (PeNSE 2019, acronym in Portuguese) ^28^, carried out with more than 125,000 students from public and private schools in the country, aged between 13 and 17 years: 45.5% of people with sexual initiation stated that they had already used emergency contraception at some point in their lives. This is, in fact, an important device in the current management of sexuality and reproduction ^29^.

The little dialogue about sex and sexuality with family members, the insufficient information on prevention, sexual and reproductive health offered by the school, the numerous contraceptive difficulties, and the gender asymmetries in the contraceptive/preventive negotiation processes are some of the elements that remain in the cases of unforeseen pregnancies. The challenge of dealing with an unforeseen pregnancy in adolescence continues to be marked by the social class of these young people and their families, leaving those who depend on state support for child care (daycare) adrift, with much more difficulties in continuing their school or work trajectories outside the home. The discomforts and side effects that hormonal contraceptives cause often imply temporary or definitive discontinuation of the method in use in search of something that interferes less with the female body ^30^ ^,^ ^31^. Contraceptive alternations open spaces for times of vulnerability ^32^ and the occurrence of pregnancy. It is important to note that many young women who even considered abortion ^33^ soon face several barriers in view of the context of illegality and moral rejection surrounding the practice of termination of pregnancy.

Finally, it is worth reflecting on the initial question that structures this research: would we be undergoing changes or permanences/continuities of certain patterns in youth behaviors? Are the continuity traits indicated by the data the effects of setbacks and breaks in processes suffered in public health and education policies in the last decade? There are issues to be faced, as representations, values and behaviors about gender and sexuality have strong dynamics of continuity despite observed changes.

Young people continue to resent the lack of frank and open dialogue and support in schools and other entities “*of the adult world*” in relation to the exercise of sexuality. The representations on external prophylactics are similar to those reported by research conducted in past decades. Contraceptive responsibilities remain assigned to women. Motherhood ends up becoming compulsory in a context of contraceptive difficulties and illegal abortion. Youth sexuality, and especially sex education in schools, have been challenged by conservative and religious leaders who have reacted to successful public policies, which are fundamental to controlling AIDS and reducing adolescent pregnancy rates. The “gender ideology” ^34^, the theses of the “non-party school” movement, have grown significantly from the 2010s ^5^, or the “I choose to wait” movement with important reverberations from the Federal Executive branch ^34^ ^,^ ^35^ neglect the right to a pleasurable sexual experience, with prevention and self-care, consensual and without violence, and build barriers to quality information.

There is youth agency in the search for information (whether related to sexuality or pregnancy or STI prevention methods). The difficulty in finding young people with children shows the trend observed in population rates on fertility and statistical projections on teenage pregnancy. Despite the challenges posed by the State itself (but also by life contexts, gender asymmetries, racial discrimination), we can assume the construction of processes of exercising sexuality more apart from the paths of reproduction in this generation.

Gender and sexuality issues have occupied a central place in the Brazilian democratic struggle. Television media, social networks and artistic and cultural segments highlight issues concerning homosexuality and feminist ideals. However, the concepts of choice and autonomy have faced the proliferation of highly restrictive and regulating discourses on sexuality. The expansion of the rights on gender identity and sexuality is confronted with initiatives to restrict pluralism led by conservative religious sectors in the legislative branch. The fight against the so-called “gender ideology” is structured in the dissemination of moral panic affirming the decay or disintegration of the family, reproduction and heterosexuality ^34^.

There is still the challenge of building less alarmist and more comprehensive perspectives on youth sexuality within the scope of public actions and policies for adolescents and young people in the country, based on care and respect for the different intersectional dimensions of young people's lives, including with regard to sexual and reproductive rights and health ^5^. It is equally challenging (and imperative) to advance in the construction of situated knowledge that can contemplate the diversity of youth life contexts and trajectories.
