# Supplementary material for: Sexuality and reproduction: contributions of multisituated socioanthropological research with “digital natives”
Source: Cad Saude Publica. 2025 May 19;41(4):e00229323. doi: 10.1590/0102-311XEN229323 (PMC12091856; doi:10.1590/0102-311XEN229323)
Supplement: Translation

# Translation: Sexualidade e reprodução: aportes de uma investigação socioantropológica multissituada com “nativos(as) digitais”

**DOI:** 10.1590/0102-311XPT229323

**Authors:** Cristiane da Silva Cabral, Elaine Reis Brandão, Flávia Bulegon Pilecco, Ana Paula dos Reis, José Miguel Nieto Olivar, Daniela Riva Knauth

**Affiliations:** 1 Faculdade de Saúde Pública, Universidade de São Paulo, São Paulo, Brasil.; 2 Instituto de Estudos de Saúde Coletiva, Universidade Federal do Rio de Janeiro, Rio de Janeiro, Brasil.; 3 Universidade Federal de Minas Gerais, Belo Horizonte, Brasil.; 4 Instituto de Saúde Coletiva, Universidade Federal da Bahia, Salvador, Brasil.; 5 Faculdade de Medicina, Universidade Federal do Rio Grande do Sul, Porto Alegre, Brasil.

**Keywords:** Jovens, Sexualidade, Gênero, Saúde Reprodutiva, Socialização

## Abstract

O artigo se debruça sobre os processos de socialização para a sexualidade entre jovens, destacando dimensões de continuidade e de mudança em relação à literatura brasileira sobre juventude produzida no início do século XXI. Entrevistamos 194 jovens de 16 a 24 anos, entre outubro de 2021 e julho de 2022, residentes em diferentes cidades e contextos territoriais (capital e interior) do Brasil, compondo um conjunto social e culturalmente diversificado, em termos raciais, de classe social, identidade de gênero, orientação sexual, com marcas distintas e acesso diferencial às tecnologias digitais e aos serviços de saúde e educação. A discussão dos resultados preliminares inclui as primeiras aproximações ao tema da sexualidade, a iniciação sexual em parceria, as dificuldades relativas à prevenção da gravidez, bem como os episódios reprodutivos. Por um lado, os resultados mostram fortes elementos de continuidades em termos de moralidades e comportamentos sexuais juvenis no processo de transição para a vida adulta de jovens brasileiros atualmente. Por outro, há no material empírico da pesquisa um uso intenso e espraiado da contracepção de emergência na maioria dos sítios investigados (exceto no Amazonas), e uma expressiva fluidez das experiências sexuais entre parcerias não estritamente heterossexuais, temas a serem ainda analisados nos próximos anos, com intuito de iluminar as peculiaridades geracionais desse processo.

## Introdução

O processo de construção de si e de transição para a vida adulta é marcado pela intensificação da socialização para a sexualidade na adolescência e juventude, compreendendo o aprendizado das normas e expectativas de gênero que modelam os comportamentos de meninos e meninas. Esse é o pressuposto geral que empresta contornos à investigação realizada entre 2021-2022, *Jovens da Era Digital: Sexualidade, Reprodução, Redes Sociais e Prevenção às IST/HIV/Aids*. Realizamos a pesquisa junto a jovens de 16 a 24 anos, homens e mulheres cisgêneros, mas também pessoas trans, de distintas inserções sociais segundo os marcadores de gênero, classe social, cor/raça, experiência reprodutiva, e residentes em diferentes regiões do Brasil. Entrevistamos jovens de distintas cidades no intuito de captar a diversidade dos processos de socialização e de sociabilidade juvenis em contextos urbanos metropolitanos e do interior. Trata-se de um empreendimento com vistas a produzirmos apreciações comparativas entre as investigações sobre sexualidade e reprodução juvenil do início dos anos 2000 e o atual contexto histórico-político-social.

Testemunhamos um espraiado uso da internet e interação sociais atravessadas pelas redes sociais. Sem dúvida, computadores e *smartphones* trouxeram mais do que uma revolução tecnológica; argumenta-se sobre uma “revolução social” ao suscitar novas relações entre as pessoas e o ambiente ^1^ ^,^ ^2^. As “redes sociais” e suas atuais formas de conexão via internet emergem como importante agente no atual cenário de sociabilidade e socialização juvenil, promovendo difusão, reificação, contestação, modulação de comportamentos, valores e práticas diversas. Pelo intermédio delas, determinados eventos ganham a luz do dia e rapidamente conectam pessoas inicialmente apartadas.

Os(As) jovens têm enfrentado profundas transformações no mundo do trabalho, mais precarizado e informal, permeados pela intensidade da violência de Estado (especialmente policial), a discriminação étnica, racial e de gênero, em momento de ascensão da extrema direita no contexto internacional e nacional, com fortes impactos no debate público sobre sexualidade e acirramento das desigualdades de gênero. Os comportamentos sexuais juvenis são atravessados pelo recrudescimento da moral conservadora no contexto sociopolítico nacional e internacional ^3^, que tem promovido uma contínua supressão ou desmonte de iniciativas de educação em sexualidade, gênero e direitos, e de programas de prevenção de infecções sexualmente transmissíveis (IST)/HIV/aids e gestação nas escolas ^4^ ^,^ ^5^.

Este estudo visa compreender os comportamentos sexuais e reprodutivos de jovens brasileiros(as), seus contextos e desdobramentos. Indagamos sobre possíveis especificidades da geração atual, com especial interesse sobre as práticas (ou ausência) de proteção diante dos “perigos advindos do sexo” ^6^ ^,^ ^7^. A reflexão sobre a maneira como os “riscos” contemporâneos associados à sexualidade são geridos no cotidiano ^8^ ^,^ ^9^ é relevante, buscando compreender as lógicas subjacentes à adoção ou não de método em determinado contexto, tanto social-cultural quanto do curso da vida. Ao que parece, os efeitos da cruzada moral conservadora e do desmonte de diversas políticas públicas na área da saúde e da educação já se fazem sentir nos resultados apresentados. Optamos pelo uso da grafia os(as) nas palavras com flexão de gênero. Trata-se de registrar a dimensão política que a linguagem comporta, buscando romper com (ou, ao menos, explicitar) a tradicional universalização dos sujeitos pela linguagem no masculino.

## Aspectos metodológicos

Estudo socioantropológico e multicêntrico, com realização de 194 entrevistas individuais, com roteiro semiestruturado, junto a jovens com idade entre 16 e 24 anos, tendo a abordagem biográfica como perspectiva teórica-metodológica para analisar contextos, elementos e repercussões de determinados eventos afetivo-sexuais-reprodutivos dos(as) envolvidos(as) ^10^.

Realizamos a pesquisa em quatro capitais - Porto Alegre (Rio Grande do Sul), Rio de Janeiro, Salvador (Bahia) e São Paulo - e em duas cidades do interior do país: Conceição do Mato Dentro, em Minas Gerais (23 mil habitantes) e São Gabriel da Cachoeira, no Estado do Amazonas (aproximadamente 51 mil habitantes). Com essa diversidade (cota de 40 entrevistas por capital e 20 em cada cidade do interior), buscamos elementos de caracterização dos valores, sociabilidade e experiências segundo contrastes regionais, nos perguntando sobre contraposições possíveis entre capital e interior.

Compusemos o conjunto de participantes de forma intencional segundo critério de cotas por sexo/gênero, inserção social e experiência reprodutiva antes dos 20 anos de idade, marco etário utilizado pela Organização Mundial da Saúde (OMS) e pelo Ministério da Saúde para identificar gestações/experiência de parentalidade na adolescência. Buscamos uma diversidade em termos de orientação e identidade sexual e racial, tendo em vista o objetivo de compreender modulações importantes nas biografias sexuais e reprodutivas a partir de dimensões interseccionais como cor/raça, classe social, identidade sexual e de gênero, região, entre outros marcadores sociais de diferenciação.

De modo a viabilizar um contexto de aproximação geracional na produção dos dados empíricos, selecionamos e treinamos pesquisadores(as) jovens, graduados(as) ou cursando a pós-graduação em Saúde Coletiva ou Ciências Sociais. Uma entrevista é uma relação social, estabelecida no encontro entre duas intersubjetividades ^11^. Não há controle dessa interação, mas se pode privilegiar os elementos necessários para uma boa qualidade deste encontro, sempre singular.

As estratégias de divulgação da pesquisa e aproximação a potenciais participantes incluíram a mobilização da rede de relações pessoais e profissionais dos(as) pesquisadores(as) envolvidos(as), circulação de textos informativos em grupos de WhatsApp e outras redes sociais (Facebook e Instagram), formulário eletrônico para identificação de jovens interessados(as) em conceder entrevistas, além do recurso à técnica de bola de neve. O trabalho de campo transcorreu entre outubro de 2021 e julho de 2022, período de amenização da pandemia de COVID-19, e possibilidade de retorno às atividades presenciais nas universidades envolvidas no estudo. As entrevistas aconteceram majoritariamente de forma presencial e em local escolhido pelo(a) participante. Todas as entrevistas contam com notas de campo, contendo descrição de onde e como o(a) entrevistado(a) foi abordado(a), a negociação e condução da entrevista em si, facilidades, dificuldades e percepções gerais sobre a interação com o(a) entrevistado(a).

A busca por jovens dispostos(as) a falar de suas trajetórias afetivo-sexuais foi difícil em todos os centros, especialmente entre homens. As quotas de entrevistadas foram as primeiras a serem preenchidas, sobretudo as de camadas populares. Inversamente, rapazes de camadas médias e com alguma experiência reprodutiva foram difíceis de serem encontrados. Especialmente em São Gabriel da Cachoeira parece ter sido ainda mais difícil poder conversar sobre sexo: diversos(as) jovens recusaram os convites, outros(as) interromperam as entrevistas, manifestando desconforto ou negativa mais ostensiva a falar sobre assuntos relacionados à sexualidade com estranhos.

Em resumo, o conjunto de jovens entrevistados(as) (n = 194) compreende mulheres cis (n = 100) e homens cis (n = 87), mas também pessoas trans (*masculines* e *feminines*) e não binárias (n = 7). Mais da metade se declarou preto(a) ou pardo(a) (n = 119); quase um terço são LGBTQIA+ (n = 58). Tem uma presença importante de jovens sem religião (n = 89), mas também católicos (n = 25) ou evangélicos (n = 30), aspecto interessante para refletirmos no futuro quanto a efeitos do pertencimento religioso sobre o comportamento sexual e reprodutivo dessa geração. Há um contingente amplo de jovens com o Ensino Médio e somente cinco jovens com baixíssima escolarização. Certamente, eles(as) existem nos grandes centros urbanos e no interior, mas estão em número cada vez menor tendo em vista o sucessivo esforço governamental de expansão do ensino de modo geral e de eliminação do analfabetismo no país. Há que se considerar igualmente esse perfil com baixa escolaridade como reflexo das estratégias utilizadas para convidar à pesquisa (convite online e bola de neve).

Gravamos e transcrevemos as entrevistas, que foram revisadas pelo(a) próprio(a) entrevistador(a). Produzimos resumos analíticos e quadros sintéticos das biografias como estratégia auxiliar para manejo do grande volume de material empírico. Usamos o software NVivo (https://www.qsrinternational.com/nvivo/home) como ferramenta de apoio para organização, categorização e armazenamento do conteúdo produzido nos seis sítios. O estudo foi aprovado pela Comissão Nacional de Ética em Pesquisa (CONEP) e pelos comitês de ética em pesquisa das instituições coparticipantes (Universidade de São Paulo: CAAE 46977021.3.0000.5421; Universidade Federal do Rio de Janeiro: CAAE 46977021.3.3005.5286; Universidade Federal da Bahia: CAAE 46977021.3.3003.5030; Universidade Federal de Minas Gerais: CAAE 46977021.3.3001.5149; Universidade Federal do Rio Grande do Sul: CAAE 46977021.3.3002.5347). Todos(as) entrevistados(as) assinaram o Termo de Assentimento Livre e Esclarecido (TALE) - houve dispensa parental de consentimento para menores de 18 anos - ou o Termo de Consentimento Livre e Esclarecido (TCLE) - jovens de 18 a 24 anos. Nomes fictícios são utilizados nesta publicação.

## Resultados

A análise em conjunto das 194 entrevistas é um desafio. Neste artigo, traçamos um panorama inicial acerca de alguns temas para compreender determinadas mudanças e permanências na vivência da sexualidade de jovens nascidos e socializados nesse período de interações por meio das redes sociais e da digitalização do cotidiano ^12^ ^,^ ^13^. Especial interesse é dado ao processo de socialização para sexualidade e aos comportamentos preventivos (ou sua ausência) em relação à contracepção e à reprodução. Os eixos estruturantes dos resultados apresentados são ^14^: agentes de socialização para a sexualidade e o início da vida sexual em parceria; a permanência das dificuldades contraceptivas; e experiência reprodutiva: episódios de gravidez e de aborto.

### Agentes de socialização para a sexualidade e o início da vida sexual em parceria

A socialização para a sexualidade advém não apenas com as primeiras informações sobre sexo, formas de prevenção de gravidez e IST, mas também com conversas sobre primeiros beijos, namoros, experiências eróticas com o próprio corpo e as experiências sexuais com parceiros(as). Trata-se de um processo que se intensifica com as mudanças corporais percebidas, no âmbito da família, da escola ou em grupos de pares ^15^.

De forma geral, os(as) jovens identificam a escola como o lugar onde obtiveram as primeiras informações sobre temas relacionados à sexualidade. Tecendo críticas, eles(as) apontaram que os conteúdos ministrados, eminentemente durante o ensino fundamental, foram centrados nos aspectos biológicos, aparelho reprodutivo e nas formas de prevenção de IST/HIV/aids, abordagem insatisfatória diante do que almejavam receber. No entanto, o tema da violência sexual e de gênero parece ter adentrado o ambiente escolar pois, em diversas narrativas, foi ressaltada a importância das discussões sobre identificação de casos de abuso e violência sexual. Apesar das críticas, as instituições de ensino aparecem como espaços seguros para se fazer questionamentos da heteronormatividade compulsória.

Outras fontes de informação são citadas como veículos para aproximações com o tema da sexualidade: televisão e filmes; internet (ferramentas de pesquisa online como Google) ou por meio de *influencers* no Instagram e no TikTok; funk; revistas eróticas; aulas de catecismo ou em grupos de jovens da igreja, entre outros. A maioria dos(as) jovens relatou acesso à pornografia de forma casual, em situações como recebimento de conteúdo não solicitado via redes sociais, mas há também busca por tal conteúdo na internet. Esses relatos apareceram de modo mais frequente em São Paulo, onde jovens indicaram assistir de forma individual ou coletiva a materiais pornográficos. 

O pânico moral que responsáveis e instituições de ensino frequentemente têm sobre possível acesso desenfreado de jovens a conteúdos pornográficos por meio da internet não ganha lastro nas entrevistas. Ao contrário, os/as jovens falam de forma crítica sobre esses conteúdos: alguns(algumas) indicaram que, após satisfeita a curiosidade em relação ao sexo, pararam de acessar vídeos pornô por não o considerarem “saudável”, por falta de interesse ou por avaliarem que a pornografia fornece uma visão estereotipada do sexo e dos corpos. Um jovem de Salvador (20 anos, classe média) afirmou que “*a pornografia é um sexo diferente da realidade*” e prejudicial para a prática sexual com parceiras reais. Esse tipo de posicionamento tem sido encontrado em outros contextos ^16^.

Poucos(as) apontaram a família como fonte das primeiras informações sobre sexualidade. O tema ainda é considerado tabu e, quando abordado, são as figuras femininas como mães, primas, irmãs que falam pelo prisma do risco do engravidamento (para mulheres) ou da infecção por IST (para rapazes). O silenciamento parece girar em torno do medo de que o diálogo sobre o assunto seja uma forma de incentivo às relações sexuais, tendo por consequência a reprodução precoce e um desvio no processo de transição para a vida adulta. Essa é uma característica que permanece indelével em mais de 20 anos de pesquisas sobre jovens e sexualidade, pois o resultado é semelhante a outros estudos do início dos anos 2000 ^17^ ^,^ ^18^ ^,^ ^19^ ^,^ ^20^. Em Conceição do Mato Dentro, uma entrevistada de família evangélica relatou que, quando tinha 11 ou 12 anos, uma professora deu um livro de Ciências no qual era abordado o tema sexualidade. Nessa ocasião, sua avó rasgou o livro, o que a fez sentir muita vergonha. Assim, ela concluiu que a família não era uma boa fonte de informações/diálogo. Poucos(as) entrevistados(as) relataram conversar com figuras masculinas sobre o tema. Um rapaz de Salvador indicou que sua mãe costumava dar conselhos, enquanto o pai era “*machista*”, como ele definiu, e lhe incentivava a transar cedo, não se preocupando com o que poderia ocorrer com as suas parceiras: “*ele* [o pai] *dizia ‘tem que pegar essas mulé mesmo, bote camisinha, faça miséria!’*”.

A iniciação sexual com parceiro(a) é um dos principais eventos no processo de socialização para sexualidade na juventude ^21^. Evento esperado, nem sempre planejado, a primeira relação sexual dos(as) entrevistados(as) ocorreu entre os 14 e 16 anos. As garotas, majoritariamente, tiveram sua “primeira vez” com garotos mais velhos ou da mesma idade; no caso dos rapazes, ela se deu principalmente com meninas da mesma idade. No Rio de Janeiro e em Salvador, os jovens citaram também, com certa frequência, a iniciação sexual com mulheres um pouco mais velhas e com mais experiência sexual. No geral, eles(as) identificaram suas parcerias como pertencentes ao mesmo grupo racial, com poucas exceções.

As especificidades da iniciação sexual de pessoas LGBTQIA+ (n = 58) não estão contempladas neste artigo. No entanto, cabe observar que a primeira relação sexual desses(as) jovens incluem tanto parceiros(as) de sexo oposto, quanto de mesmo sexo (nesse caso, 6 mulheres e 9 homens). Também estão ausentes as particularidades dos(as) jovens virgens (n = 28): eles(as) têm entre 15 e 23 anos, majoritariamente sem religião (n = 15), pessoas cisgênero (n = 27).

A primeira relação sexual é majoritariamente aquela que envolveu sexo com penetração, geralmente descrita como desconfortável e ruim. Uma jovem (São Paulo) indicou que a relação não tinha sido prazerosa, afirmando que “*era como se eu estivesse só pra dar prazer e não pra receber*”. Olívia (Conceição do Mato Dentro) descreve sua iniciação sexual como “*traumatizante*”, fruto de um impulso. Várias jovens no Rio de Janeiro afirmaram que sentiram dor, foram pressionadas ou que o parceiro foi “*machista*”. O relato de um jovem (Rio de Janeiro) que viveu a primeira relação com a namorada disse que ela tinha uma expectativa maior do que realmente foi: “*Ela esperava uau e foi somente sexo mesmo*”.

Há também relatos da iniciação sexual como algo bom e satisfatório. Luana (20 anos, classe popular, Salvador) gostou da sua primeira relação sexual, apesar de não ter sido “*romântica*”. Ela tinha 15 anos e o parceiro 18 (ele é seu atual parceiro e pai de sua filha). O sexo aconteceu na casa dele quando os familiares estavam fora, a mãe, trabalhando e a irmã mais nova, na casa de uma prima, “*não foi nada forçado... foi por curiosidade e vontade*”. Embora a maioria dos(as) jovens classifique a primeira experiência sexual como ruim, é comum o relato de que as experiências seguintes, com parceiros(as) com quem têm mais intimidade, são melhores, pois começam a aprender sobre prazer, conhecem melhor seu corpo e o do(a) parceiro(a).

A iniciação sexual é majoritariamente descrita como algo que “*aconteceu*”, guardando contornos de certo tipo de espontaneidade, aspecto outrora sinalizado como estruturante do início da vida sexual de jovens brasileiros(as) ^21^. Ainda que minoritários, há casos com planejamento prévio, algo que foi “*cozinhado*” em parceria. Ela tem lugar no contexto doméstico, na casa de uma das duas pessoas envolvidas. Algumas jovens (Conceição do Mato Dentro e São Paulo) afirmaram que a primeira vez foi discutida previamente com seus namorados, em relação de “*confiança*” e “*carinho*”. Em Conceição do Mato Dentro, as garotas que indicaram ter planejado o evento tiveram sua iniciação sexual em idade um pouco mais avançada do que as outras meninas do mesmo sítio, que avaliaram sua iniciação sexual de forma mais negativa.

A linguagem empregada é reveladora da moralidade sexual e de gênero que organiza as interações sexuais. Apesar das diversas mudanças relativas à autonomização dos corpos e da sexualidade feminina, ainda permanecem termos tradicionais para descrever a iniciação sexual das moças. Por exemplo, no Rio de Janeiro, algumas jovens de camada popular se referiram à primeira experiência como tendo “*se perdido*”: “*Logo assim que eu me perdi eu contei para minha mãe*”. Em São Gabriel da Cachoeira, o termo acionado é “*se guardar*” para designar o contexto em que, após a única relação sexual tida, em torno dos 14/15 anos na casa dela com um “*ficante*”, a entrevistada afirma que “*se guardou*” novamente, para continuar seus estudos. Em um contexto, perder a virgindade expressa uma conotação moral que subtrai da jovem algum atributo de valor, que ela teria perdido ao “*se entregar ao parceiro*”. No outro contexto, “*se guardar*” parece remeter a uma estratégia de vida e tentativa de controle sobre a reprodução diante da falta de preparo ou de recursos para evitar gravidez ou IST.

### A permanência das dificuldades contraceptivas

A contracepção permanece sendo uma responsabilidade das mulheres, mesmo em contexto em que o uso do preservativo externo foi mencionado de modo recorrente. As jovens indicaram principalmente a internet, com realização de buscas no Google, como fonte de informação sobre métodos contraceptivos. Algumas moças assistiram a vídeos no YouTube; outras afirmaram seguir perfis de médicas ginecologistas no Instagram, acompanhando conteúdos sobre contracepção. No caso dos homens, percebeu-se um desconhecimento generalizado em relação aos contraceptivos femininos e seus mecanismos de ação.

O preservativo externo e o coito interrompido são usados de forma intercalada a métodos hormonais como estratégia para evitar gravidez. O preservativo interno apareceu ocasionalmente nos relatos de jovens de São Paulo e Rio de Janeiro. No entanto, o preservativo continua sendo um artefato incômodo para essa geração. Os esquivos e o desconforto aparecem tanto nas narrativas de iniciação sexual quanto nos encontros subsequentes. Apesar das dificuldades, o provisionamento do insumo esteve majoritariamente a cargo dos rapazes, por meio das farmácias privadas e, em menor proporção, nas unidades básicas de saúde (UBS).

Entre os métodos hormonais, a pílula anticoncepcional é predominante em algum momento das trajetórias sexuais das jovens. O contraceptivo injetável, em sua maioria, usado por moças de camadas populares, alterna-se com outros métodos comportamentais (coito interrompido; tabelinha) ou de barreira. Foi difícil captar alguma regularidade na utilização de métodos contraceptivos; a alternância de parcerias ajuda nessa inconstância, assim como a busca por encontrar um método satisfatório. Jovens menores de 18 anos relataram dificuldades de acesso a métodos diferentes da camisinha externa, situações em que era demandada (indevidamente) o acompanhamento adulto durante o atendimento ginecológico, ou, ainda, falta de dinheiro para acessar outros métodos contraceptivos nas farmácias privadas.

Esquecimento na reposição dos contraceptivos e desconhecimento do uso correto estão subjacentes a algumas experiências reprodutivas imprevistas. Impressiona encontrar, na geração atual, relatos comumente obtidos décadas atrás. Exemplos: uma jovem de Salvador (20 anos, com gravidez aos 16 anos) decidiu começar a tomar a pílula, a mesma que a mãe de sua amiga usava. Contudo, a moça utilizava de forma errada, tanto o anticoncepcional como a pílula do dia seguinte: “*Toda vez que eu tinha relação eu ia lá e tomava um remédio, e achava que tava de boa. Aí depois eu comecei a tomar a pílula do dia seguinte, não tomava todo dia, mas tomava com muita frequência*”. Uma outra jovem (São Gabriel da Cachoeira) narrou que sua mãe a orientava a tomar “*vacina*” para prevenir gravidez; no entanto, ela utilizava camisinha de vez em quando e às vezes tomava contraceptivo oral. Tinha medo de que algo acontecesse com seu corpo e parou de tomar o “*remédio*” - encontrava-se grávida no momento da entrevista.

A relação com profissionais da saúde, desde as narrativas das jovens, deixa muito a desejar do ponto de vista do acolhimento necessário, do diálogo sobre os métodos contraceptivos e esclarecimento de dúvidas. Em geral, as jovens conversam com amigas sobre contracepção. Escolas e serviços de saúde aparecem muito pouco como espaços de debate ou de fonte de informação sobre o tema.

Boa parte das jovens não estão satisfeitas com os métodos contraceptivos em uso e anseiam por um método que lhes tragam maior conforto e menos efeitos colaterais. É bastante significativo a quantidade de sintomas e efeitos colaterais provocados pelos métodos hormonais, prejudicando sensivelmente o cotidiano das jovens. Naquelas que utilizam pílulas anticoncepcionais ou injetáveis há muitas queixas de sangramentos excessivos, dores de cabeça, inchaço/edemas, ganho de peso, problemas vasculares, mal-estar, indisposição e diminuição da libido. A alternância entre tipos de métodos foi bastante frequente, havendo uma composição entre eles ao longo das trajetórias juvenis. Essas tentativas de se ajustar a alguma proteção que seja menos iatrogênica abrem a possibilidade de ocorrência de gravidez imprevista.

Vale mencionar a presença de informações em São Gabriel da Cachoeira sobre “*benzimentos*” e processos rituais associados à menarca e à prevenção da gravidez nas narrativas de jovens. Em rodas de conversa mantidas durante o trabalho de campo, diversas jovens explicaram de forma consistente a importância - e eficácia, quando “*bem feitos*” - desses procedimentos ancestrais que não são apenas para prevenir uma gravidez, mas para cuidar do corpo, da vida, dos conhecimentos e das possibilidades reprodutivas das mulheres.

### Experiência reprodutiva: episódios de gravidez e de aborto

A busca por participantes com algum evento reprodutivo na trajetória foi intencional. Esperávamos reunir 100 pessoas (metade do universo empírico), mas houve muita dificuldade para identificar jovens com esse perfil. Conseguimos entrevistar 83 pessoas, com 98 episódios reprodutivos, grande maioria sendo levado a termo. Registramos apenas 11 abortos (cinco provocados). Há 15 entrevistados(as) com mais de uma gravidez.

De modo geral, os relatos indicam gestações que ocorreram de forma imprevista. Os contextos de planejamento aludem à gravidez como estratégia para sair da casa dos pais e/ou se afastar da família de origem (marcadas por forte vulnerabilidade social). Uma jovem de 17 anos, preta, de classe popular, moradora de Porto Alegre, considera que sua vida está mais fácil agora que é mãe e reside com o marido. Antes ela cuidava de suas duas irmãs mais novas para sua mãe trabalhar, e acredita que já tenha vivido uma experiência de maternidade nesse período: “*mãe das minhas irmãs*”. Em geral, os(as) entrevistados(as) relataram que houve um entendimento entre o casal sobre a manutenção da gravidez. A maioria das jovens mães afirmou ter contado com o apoio do parceiro e da família no decorrer das gestações e após o parto com os labores de cuidado com a criança. A coabitação após a descoberta da gestação ou do parto foi frequente em quase todos os casos, ainda que em alguns tenha ocorrido uma posterior separação.

Muitas entrevistadas disseram ter pensado na realização do aborto. Porém, fatores como o *status* de ilegalidade do procedimento, falta de apoio do parceiro e da família, questões religiosas e morais pareceram influenciar a decisão de levar as gestações a termo. Laíz (Conceição do Mato Dentro), 14 anos à época, classe popular, católica, parda, relata que queria interromper a gravidez logo que a descobriu, mas ouviu que ela “*teria que assumir o seu erro*”. Uma jovem do Rio de Janeiro foi obrigada pela mãe a levar a gestação adiante. No Rio de Janeiro, apenas uma moça e um rapaz falaram sobre interrupção de gravidez. Em ambos os casos, as famílias das moças sabiam das gestações e apoiaram a decisão pelo aborto; a família do rapaz não teve conhecimento da gravidez. Uma jovem em São Paulo, grávida aos 18 anos, decidiu pelo aborto com apoio de seu parceiro, que comprou o medicamento Cytotec pelo Facebook. O procedimento aconteceu na casa dele. Em São Gabriel da Cachoeira, uma entrevistada conta que, perante a suspeita de gravidez devido a um atraso menstrual, ingeriu grandes quantidades de chá e sua menstruação “*desceu*”. Em suas palavras: “*chá de boldo salva muita gente*”.

## Discussão

Os atuais dados empíricos e reflexões decorrentes sobre socialização para sexualidade, iniciação sexual (parecerias heterossexuais), práticas contraceptivas e reprodução, incluindo o aborto, entre jovens de 16 a 24 anos, têm como principal contraponto as produções do início dos anos 2000 sobre jovens brasileiros ^18^. Essa é uma estratégia analítica que busca refletir sobre possíveis mudanças e permanências/continuidades quanto aos comportamentos sexuais e reprodutivos e moralidades juvenis, após duas décadas de transformações nas sociabilidades dos(as) jovens, permeadas por drásticas mudanças políticas, econômicas, ambientais e digitalização do cotidiano ^12^ ^,^ ^13^ ^,^ ^16^.

Essa geração de jovens, por vezes denominada como “nativos digitais”, permeada pela consolidação e super massificação das comunicações online, também foi interpelada pela ocorrência da pandemia de COVID-19. A pesquisa e as vidas juvenis foram atravessadas pela primeira grande emergência sanitária do século XXI. Muitos(as) jovens navegaram em meio ao medo da infecção, o maior/menor controle parental e as alternativas inventadas para recuperação da sociabilidade no período imediatamente anterior ao trabalho de campo empreendido.

A iniciação sexual continua sendo marcada por certo espontaneísmo ^21^, com poucos casos de planejamento, sobretudo anticonceptivo, à primeira relação sexual. A moralidade sexual em termos de “*perda da virgindade*” segue presente na atual geração. A primeira relação sexual continua sendo considerada majoritariamente aquela em que há sexo com penetração ^22^.

Antigos e novos atores habitam o cenário do aprendizado da sexualidade. Família e escola permanecem como importantes agentes no processo de socialização às práticas contraceptivas e preventivas: as mães são referência para moças e rapazes, ainda que as conversas não sejam francamente abertas e tenham contornos de “*tomem cuidado*”; os pais seguem ausentes nesse suporte. As escolas, por seu turno, continuam em descompasso com as expectativas juvenis. Os temas relativos ao domínio da sexualidade estão acantonados nos conteúdos escolares, abordados sob a forma da responsabilização individual e do risco (sexo como perigo) em diversas escolas nas diferentes cidades estudadas.

Há novos atores em cena: a internet é fonte de informações diversas para os(as) jovens, inclusive sobre sexualidade. Ela multiplica as possibilidades de sociabilidade, de acesso a informações e conhecimentos os mais diversos. Enquanto “nativos digitais”, o que certamente não se aplica da mesma forma em todos os contextos, esses(as) jovens aprendem a navegar o mundo que habitam, produzindo caminhos e conhecimentos em seus processos de socialização para a sexualidade.

Uma diferença considerável entre os(as) jovens das cidades estudadas foi a forte recusa a falar sobre sexualidade e saúde reprodutiva em São Gabriel da Cachoeira, o que reverberou em dificuldades na condução das entrevistas acerca de determinados temas e detalhamentos solicitados. No entanto, essa recusa parece não ser atinente apenas ao contexto da entrevista. Conversas entre jovens e suas mães geralmente tratam do contexto da menarca - “*cuidados*” a serem adotados no período menstrual. Diálogo sobre desejos e práticas sexuais são possíveis entre pares, em contextos íntimos, protegidos e cotidianos, particularmente entre moças ^23^. Observações etnográficas sugerem que dentro da dinâmica local, conversar sobre sexualidade em relações cruzadas de gênero e, especialmente, com diferença etária, é um ato que pode ser conectado diretamente com as dinâmicas de sexualidade e conjugalidade: a cena de um homem “*conversar*” com a moça ou com o pai dela pode implicar abertura para o sexo e para o casamento ^23^ ^,^ ^24^ ^,^ ^25^.

Não faz parte do escopo deste artigo analisar os processos que levam à queda na taxa de fecundidade adolescente em anos recentes ^26^ ^,^ ^27^. No entanto, ela se fez sentir sobremaneira em nossa investigação pois, como salientado, foi difícil encontrar jovens que tivessem tido filhos antes dos 20 anos de idade, sobretudo rapazes de camadas médias. Consideramos que o uso espraiado da contracepção de emergência entre os(as) entrevistados(as) esteja contribuindo para menor ocorrência de gestações imprevistas. Essa perspectiva é reforçada pelos dados da última *Pesquisa Nacional sobre Saúde do Escolar* (PeNSE 2019) ^28^, realizada com mais de 125 mil estudantes de escolas públicas e privadas do país, com idade entre 13 e 17 anos: 45,5% das pessoas com iniciação sexual afirmou que já havia usado o contraceptivo de emergência em algum momento da vida. Esse é, de fato, um importante dispositivo na atual gestão da sexualidade e da reprodução ^29^.

O pouco diálogo sobre sexo e sexualidade com familiares, a insuficiente informação sobre prevenção, saúde sexual e reprodutiva ofertada pela escola, as inúmeras dificuldades contraceptivas e as assimetrias de gênero nos processos de negociação contraceptiva/preventiva são alguns dos elementos que permanecem nos enredos das gestações imprevistas. O desafio do enfrentamento de uma gravidez imprevista na adolescência continua sendo bastante marcado pela inserção social destes(as) jovens e suas famílias, deixando aqueles(as) que dependem do apoio do Estado para os cuidados infantis (creches) à deriva, com muito mais dificuldades para continuarem suas trajetórias escolares ou de trabalho fora do lar. Os desconfortos e efeitos colaterais que os contraceptivos hormonais provocam muitas vezes implicam interrupções temporárias ou definitivas do método em uso em busca por algo que interfira menos no organismo feminino ^30^ ^,^ ^31^. As alternâncias contraceptivas abrem espaços para momentos de vulnerabilidade ^32^ e ocorrência de gravidez. Importante dizer que muitas jovens que chegaram a cogitar o aborto ^33^ logo se deparam com diversas barreiras tendo em vista o contexto de ilegalidade e rechaço moral que cerca a prática da interrupção da gestação.

Por fim, cabe refletir sobre a inquietação inicial que estrutura essa investigação: estaríamos diante de mudanças ou de permanências/continuidades de determinados padrões nos comportamentos juvenis? Os traços de continuidade que os dados apontam são efeitos de retrocessos e quebras de processos sofridos nas políticas públicas de saúde e de educação da última década? Há tensões a serem enfrentadas, pois representações, valores e comportamentos sobre gênero e sexualidade abrigam fortes dinâmicas de continuidade a despeito de mudanças registradas.

Os(As) jovens seguem ressentidos(as) pela ausência de diálogo franco e aberto, de suporte em escolas e demais entidades “*do mundo adulto*” em relação ao exercício da sexualidade. As representações em torno do preservativo externo são semelhantes às retratadas por pesquisas realizadas em décadas passadas. As responsabilidades contraceptivas seguem sendo femininas. A maternidade acaba se tornando compulsória em um contexto de dificuldades contraceptivas e ilegalidade do aborto. A sexualidade juvenil e, em especial, a educação sexual nas escolas, têm sido interpeladas por lideranças conservadoras e religiosas que reagiram a políticas públicas bem-sucedidas, fundamentais para o controle da aids e diminuição dos índices de gravidez na adolescência. A “ideologia de gênero” ^34^, as teses do movimento “escola sem partido” que cresceram sobremaneira a partir de 2010 ^5^, ou do movimento “eu escolhi esperar” com reverberações importantes desde o Poder Executivo federal ^34^ ^,^ ^35^ negligenciam o direito à experiência sexual prazerosa, com prevenção e autocuidado, consensual e sem violência, e constroem barreiras para acesso à informação qualificada.

Há agenciamentos juvenis na busca por informações (seja relativa à sexualidade ou forma de prevenção de gravidez ou IST). A dificuldade em encontrar jovens com filho atesta a tendência retratada em taxas populacionais sobre fecundidade e projeções estatísticas sobre gravidez na adolescência. Apesar das barreiras que emanam do próprio Estado (mas também dos contextos de vida, das assimetrias de gênero, das discriminações raciais), podemos supor a construção de processos de exercício da sexualidade de forma mais apartada dos caminhos da reprodução nessa geração.

As questões de gênero e sexualidade têm ocupado lugar central na luta democrática brasileira. As mídias televisivas, as redes sociais e os segmentos artísticos e culturais destacam temáticas concernentes à homossexualidade e ao ideário feminista. Mas os ideários de escolha e autonomia têm enfrentado a proliferação de discursos altamente restritivos e reguladores da sexualidade. A ampliação dos direitos das identidades de gênero e sexualidade confronta-se com iniciativas de restrição ao pluralismo providas por setores conservadores religiosos na esfera legislativa. O combate à chamada “ideologia de gênero” estrutura-se na disseminação de pânicos morais que afirmam a decadência ou esfacelamento da família, da reprodução e da heterossexualidade ^34^.

Segue o desafio de construir perspectivas menos alarmistas e mais compreensivas da sexualidade juvenil no âmbito das ações e políticas públicas para adolescentes e jovens no país, baseadas no cuidado, acolhimento e respeito às diferentes dimensões interseccionais da vida dos(as) jovens, inclusive no que se refere aos direitos e à saúde sexual e reprodutiva ^5^. É igualmente desafiante (e imperativo) caminhar no sentido da construção de um conhecimento situado, e que possa contemplar a diversidade dos contextos de vida e trajetórias juvenis.
